# The Impact of Body Posture on Heart Rate Strain during Tree Felling

**DOI:** 10.3390/ijerph191811198

**Published:** 2022-09-06

**Authors:** Petros A. Tsioras, Mahmoud Khooshdohbat, Mehrdad Nikooy, Ramin Naghdi, Mahmoud Heidari

**Affiliations:** 1 Lab of Forest Utilization, School of Forestry and Natural Environment, Aristotle University of Thessaloniki, 54124 Thessaloniki, Greece; 2 Forestry Department, Faculty of Natural Resources, University of Guilan, Sowmeh Sara 96196-43619, Iran; 3 Department of Occupational Health, School of Health, Guilan University of Medical Sciences, Rasht 41446-66949, Iran

**Keywords:** chainsaw, heart rate during work, relative heart rate index, forest operations, ergonomic interventions, motor–manual wood harvesting, Iran

## Abstract

Tree felling is recognized as one of the most difficult and physically demanding work phases in motor–manual wood harvesting, during which maintaining good posture can avert unnecessary loadings to the spine and the consequent musculoskeletal disorders to forestry professionals. This study aimed to (a) quantify the impact of posture selection by means of heart rate measurements and (b) analyze its interactions with the anthropometric and personal information of study subjects. Thirteen forest workers were asked to fell thirty trees in each of the four most common body postures during motor–manual forest operations: (i) stooping, (ii) flexed stooping, (iii) squatting, and (iv) half kneeling. Posture had a significant impact on the amount of heart strain measured as mean heart rate during work (HR_work_), heart rate increase over resting heart rate (ΔHR), and relative heart rate index (HRR). The most popular position among the forest workers was flexed stooping, which also caused the most damage, compared with the least physiologically damaging position, half kneeling: HR_work_ by 12.40 bpm, ΔHR by 10.24 bpm, and HRR by 11.51. On the contrary, overweight and older subjects experienced lower heart rate strain, a finding that has to be further investigated.

## 1. Introduction

Wood harvesting is among the most dangerous and strenuous professions in all fields of production [1,2]. Motor–manual wood harvesting, whether partially or fully mechanized, imposes high demands on the forestry workforce [3,4,5,6,7,8]. Working in remote areas on steep terrain [4] with exposure to often adverse weather conditions [9] explains the limited attractiveness of forest professions and the consequent reduction in employment numbers [10,11]. Additionally, the high prevalence of musculoskeletal disorders (MSD) [12,13,14,15,16] and high accident rates [1] underline the need for ergonomic research in forest operations as a necessity, rather than as an improvement factor in terms of productivity for the safety and well-being of the forestry workforce.

Motor–manual forest operations are physically very demanding, to a large extent due to chainsaw use. Forest workers navigate and carry and operate their equipment in difficult terrain. During their working hours, chainsaw operators are exposed to vibration [17,18], noise [19,20,21,22], dust [23,24], and exhaust fumes [25,26]. Another problem is the frequent adoption of bad body postures during chainsaw operation, a multifactorial issue [27] that unites topographic features, type and size of equipment, previous vocational training record, and anthropometric features to frequently arrive at the rather simplistic yet at the same time deeply rooted in forestry practice saying that “no two trees can be harvested in the same way”. In short, a wide variability of working postures can be identified in forest operations [2,28].

To better understand the importance of proper body posture during work, we have to consider the mechanism behind posture. What can be defined as good posture assumes a “neutral” position of the spine: not too rounded forward and not arched back too far. However, due to the inherent complexity of chainsaw operation, operators are forced to adopt awkward postures that demand muscle tension. Working muscles need appropriate amounts of oxygen and energy substrates delivered by the circulatory system [29]. Thus, postures that require a greater involvement of muscles are characterized by a greater physiological strain [28,30]. Epidemiological studies have shown that the interactions between inappropriate postures and movements, high forces, and high repetition rates can give rise to musculoskeletal disorders of various severities [31].

There is a rather limited body of literature describing the impacts of postural status on the strain experienced by chainsaw operators. This can be partially explained by different research areas of focus (for instance, measurements of physiological demands not strictly connected with specific postures) but also by the hesitation of forest workers to participate. To our knowledge, only Grzwiński et al. [28] studied the same topic, although the tree felling was simulated. Specifically, no trees were felled; rather, thin slices of wood were cut from higher than normal stumps. A total of 13 experienced forest workers participated in the present study, and each cut 30 slices in each of 4 postures. In recent research based on heart rate (HR) measurement during chainsaw operation, researchers have reported high physiological strain, with average HR in the range of 110–138 beats per minute (bpm) [3,5,28,32]. Research has shown that tree felling and processing are the most demanding phases of harvesting trees [3,28]. It comes as no surprise that chainsaw operators suffer from high MSD prevalence [2,15,33,34].

Despite a rekindled interest in occupational health issues related to forest operations during the last decade, only a few investigations have focused on the effects of posture on the physiology of chainsaw operators. The goal of this study was to assess the impacts of the four most frequently used postures during tree felling on physiological strain in chainsaw operators measured according to their heart rates. In particular, the study aimed to (a) quantify the impact of posture selection based on heart rate and (b) analyze its interactions with the anthropometric and personal information of study subjects. This study aims to contribute to the better understanding of working posture during tree felling and to propose correctional measures for the benefit of the workforce.

## 2. Materials and Methods

### 2.1. Study Area and Equipment

This study was performed in 35-year-old poplar forests in the west of Guilan Province in northern Iran (Table 1). Following the management plan, ten-year clear cut felling operations began in 2020 and will continue until 2030. The total harvest area is 351 hectares, of which 45 hectares were harvested in the first year (2020–2021) by the Aria Bishe Consulting Forest Engineering Company, which is responsible for harvesting and reforesting this area. All trees were felled with a Stihl chainsaw model MS780 (5.6 kW and 65 or 73 cm chain).

### 2.2. Participant Selection

A total of 13 apparently healthy male forest workers were chosen for measuring the physiological workload during tree felling at four postures. The inclusion of female forest workers was not possible as the profession is male dominated in the area. All subjects were selected randomly from a larger pool of volunteers representative of the forestry workforce in the area. The following criteria for participation were set: (a) in good health, (b) of different ages, (c) of different BMIs, (d) able to operate the chainsaw in all four postures under examination, and (e) comfortable using the measuring instruments. Some forest workers were excluded from participation, in most cases for not fulfilling the fourth selection criterion. None of them had previously received any kind of specialized vocational training because such training is so far not available in Iran.

The study protocol was approved by the Committee of Ethics of the University of Guilan. The workers who participated in the study were previously informed of the study goals and protocol and the data anonymization, for which their written consent was requested. Their participation was voluntary, and they could withdraw at any time. Additional personal data were collected from each participant regarding their age, work experience, and any health problems that could have excluded them from participating in our study. We measured height with a stadiometer and measured weight using a digital calibrated scale (precision of 100 g). Based on these measurements, we also calculated the body mass index (BMI) for each worker. BMI was defined as the subject’s weight in light clothing (in kilograms) divided by the subject’s squared height without shoes (in meters). According to their BMI, subjects were divided into four classes: low weight (<18.5), normal weight (18.5–24.9), overweight (25–29.9), and obese (>30) [35,36].

### 2.3. Examined Body Postures

**Figure 1:**
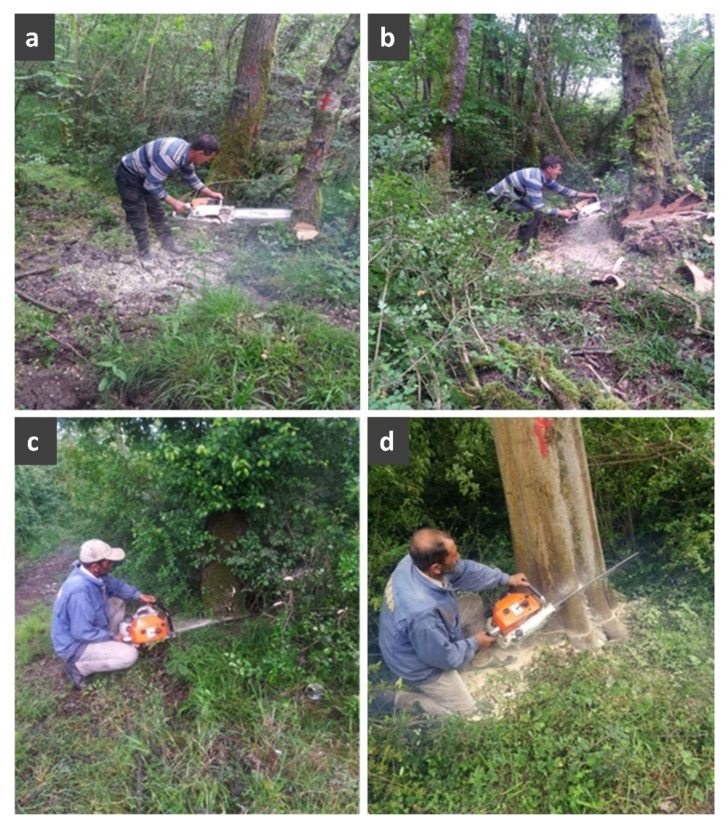
The examined body postures: (**a**) back bent forward with straight legs (stoop); (**b**) back bent forward with bent legs (flexed stoop); (**c**) squatting (squat), and (**d**) kneeling on one knee (half-kneel).

Tree felling includes the following work elements: (a) undercut, which results in an open-faced notch on the tree stem and is very important for the felling direction; (b) backcut, which is made on the opposite side of the notch and practically disrupts the continuity of the wooden tissue with the exception of a hinge that keeps the tree standing; and (c) wedging, which helps break the hinge and initiates the tree’s falling to the ground. Out of a large number of possible working postures during tree felling, we examined the four most common (Figure 1): (a) stoop, during which the back is bent forward with straight legs; (b) flexed stoop, during which the back is bent forward and the legs are also bent; (c) squat, during which the body’s weight is supported by the feet but the knees and hips are bent; and (d) half-kneel, during which the subjects kneel on one knee. The squatting position is differentiated by having a greater than 90° angle bend at the knee joint than with the other postures, where the specific angle ranged from 45 to 60° [28]. These four postures were described and examined by Grzywiński et al. [28], and our inspections of the harvesting sites of the study area justified their selection for the purposes of our study.

### 2.4. Heart Rate Measurements

The data collection lasted for one month (May 2020) at a mean temperature of 29.7 °C and a mean humidity of 76.3%. These climatic conditions are typical for this location at this time of the year. Polar Electro H10 (Polar, Finland) heart rate monitors were used during this study. Once the sensor is installed into the worker’s chest, the device samples the heart rate every 30 s and broadcasts it through a Bluetooth^®^ adapter to a Polar application (Polar beat) installed on a mobile phone. Each day, 2 out of the 13 pre-selected forest workers were randomly chosen for data collection for 2 time periods (sessions) of 2 h each, from 8–10 am and 10–12 am. Upon arrival at the study site, the heart rate monitor was attached to the subjects. A member of the research team described the procedure and answered the subjects’ questions. This period of 10–15 min also facilitated the familiarization of the subjects with the equipment. Prior to starting work, each subject was asked to sit quietly for another 15 min during which he could not talk, eat, or smoke so that his heart rate during resting (HR_rest_) could be measured. The forest workers were not given any special instructions regarding their work habits.

Each selected worker was asked to fell 30 trees in 1 of the 4 randomly selected postures. There was no interaction or visual contact between the subjects as the harvesting sites were not adjacent. After they finished felling the trees, the workers rested for about 15 min, during which they could consume sufficient water and food, and then they continued tree felling in a second, also randomly selected, posture. Two days were needed to complete the required number of tree fellings per subject (2 days × 2 postures per day × 30 trees per posture). The time study included only the sum of the time requested for the undercut and back cut work phases, including wedging, when necessary. As the data collection was carried out in a poplar plantation, neither the almost identical each time distance between the felled trees nor the minor topographical changes were considered factors that violated the validity of the measurements.

A total of 8783 HR_work_ measurements were analyzed for all workers during the data collection. The transmitted data included the heart rate of the worker during the measurement period.

The data analysis aimed at the estimation of the relative heart rate (HRR) at work according to Equation (1) [37,38]:

(1) $$\mathrm{HRR}=\frac{\left({\mathrm{HR}}_{\mathrm{work}}-{\mathrm{HR}}_{\mathrm{rest}}\right)}{\left({\mathrm{HR}}_{\max}-{\mathrm{HR}}_{\mathrm{rest}}\right)}\times 100$$

where HR_work_ was the average heart rate during work, HR_rest_ was the resting heart rate, and HR_max_ was the maximum heart rate calculated for each subject by subtracting his age from 220. The validity of 220—age has been questioned for containing a degree of error [33,39]; however, it has been widely used in applied situations as is [37,40,41].

HRR at work is an indicator of the magnitude of the operator’s physiological reserve that is used throughout a task. Most studies investigating the physiological workload of forest workers have used HRR as the main reference [42]. HRR values in the 30–40% aerobic capacity indicate a moderate physiological workload [5], allowing to the subject to work for an eight-hour period without becoming weary.

Çaliskan and Çaglar (2010) [5] compiled a physical work classification table (Table 2) that was used to determine workload levels in this investigation. The comparison of the collected data with the corresponding heart rate ranges per effort level, as shown in Table 2, allowed for this identification.

### 2.5. Statistical Analysis

Data analysis was performed with SPSS Ver. 23. Initially, the data were checked for normality with the Shapiro–Wilks test and homogeneity of variance with Levene’s test. One-way ANOVA was used to identify statistical differences among the four postures in terms of: (i) average tree diameter at breast height (DBH), (ii) time consumption during tree felling, and (iii) heart rate measurements and indices. Significant differences were further examined by with Bonferroni post hoc test with the level of significance set to α = 0.05.

General linear models (GLM) were built to detect the effects of the subjects’ anthropometric and personal information, and posture on (i) HRwork, (ii) the resulting increase from the resting heart rate (ΔHR), and (iii) the relative heart rate (HRR). Fixed factors included posture (Posture 1: Stooping; Posture 2: Flexed stoop; Posture 3: Squat; Posture 4: Half-kneel), Age class (Age class 1: age ≤ 44.62 years; Age class 2: age > 44.62 years) and BMI class (BMI class 1: BMI ≤ 25, BMI class 2: BMI > 25) of the subjects. Continuous variables that were treated as covariates included the number of work experience in years (Experience), the diameter at breast height (DBH) in cm, and time consumption for tree felling (Time) in minutes.

Outliers were initially checked in the data. To investigate possible interaction effects on the dependent variables, full factorial models were created. The goodness of fit of the regression models was tested using F-tests, while the significance of the model coefficients was tested using t-tests. In a second step, unimportant components were eliminated in order to develop reduced models for the dependent variables’ prediction. The normality and homoscedasticity of the models were graphically validated.

## 3. Results

### 3.1. Subject Anthopometric and Personal Information

The anthropometric and personal information of the examined subjects are presented in Table 3. The subjects had a mean age of 44.62 years, a mean height of 170 cm, a mean weight of 74.70 kg, and a mean BMI of 25.68 kg m^−2^ (Table 3). Six participants (46.1%) had a normal weight, six (46.1%) were overweight, and only (7.8%) was obese. Most were fairly experienced, with a mean working experience of 18.08 ± 5.16 years, ranging from 9 to 29 years.

### 3.2. Tree Diameter and Time Consumption

A total of 1560 trees were felled during the data collection period (Table 4). The average tree DBH per worker and posture ranged from 28.73 to 36.83 cm, although the grand means were almost the same among postures, ranging from 32.34 to 32.72 cm (F = 0.377, df = 3, *p* = 0.984). Tree felling demanded on average from 2.50 to 3.34 min. The grand mean of felling time ranged from 2.78 to 2.87 min and did not differ significantly among postures (F = 0.608, df = 3, *p* = 0.613).

### 3.3. Heart Rate Measurements

A total of 8783 heart rate measurements were taken during the tree felling operations (Table 5). A large range in HR extending from 104 bpm (Squat posture) to 137 bpm (Flexed-stoop posture) was found. Significant differences among workers were found in all postures (Stoop: F = 70.358, df = 12, *p* < 0.001; Flexed stoop: F = 105.093, df = 12, *p* < 001; Squat: F = 211.544, df = 12, *p* < 0.001; Half-kneel: F = 86.583, df = 12, *p* < 0.001). The grand means including all heart rates measured per posture also differed significantly (F = 4296.215, df = 3, *p* < 0.001), with the highest grand mean found for flexed-stoop posture (125.83 bpm), followed by stooping (117.04 bpm), squatting (115.16 bpm), and finally, half-kneeling (112.95 bpm).

Flexed stooping exerted heart strain equivalent to very heavy work at 53.91%, and 12 of the 13 subjects belonged to this strain category (Table 6). This group’s HRR was significantly higher than that of the other postures (F = 25.508, df = 3, *p* < 0.001). On the contrary, stooping had a grand mean of 45.5% (HRR of two subjects > 50), squatting of 43.92% (HRR of one subject > 50), and half-kneeling of 41.32% (six subjects < 40).

### 3.4. GLM Analysis of Variance and Prediction Models

Posture is the only factor affecting HR_work_ (Model 1) (Table 7 and Table 8). Flexed stooping results in considerable heart rate increases compared with the other postures. In rank of magnitude, flexed stooping is followed by stooping, squatting, and half-kneeling. In the cases of both ΔHR (Model 2) and HRR (Model 3), their predicted outcome was largely determined by subject posture type, BMI, age class, work experience (Experience), and time consumption during tree felling (Time) according to the partial η^2^ values. As the two models share the same structure, similar results were found: ΔHR and HRR increased along with work experience and time consumption during tree felling. Posture type also affected the heart rate strain, following, in terms of magnitude, the same order as in Model 1. Interestingly, younger (Age class 1) and thinner (BMI class 1) subjects evidenced greater heart rate strain than their older (Age class 2) and heavier (BMI class 2) colleagues.

## 4. Discussion

### 4.1. Heart Rate Measurements

The heart rate during the resting phase is crucial since it is utilized to calculate various heart rate indices such as the HRR. In this investigation, the mean heart rate during resting time was 68.48 bpm, which is similar to the 68 bpm reported by Abeli and Malisa [43] and close to the 70.46 bpm reported by Arman et al. [3]. Melemez and Tunay [44] recorded 70.5 bpm, while Grandjean [45] reported 72.7 bpm. On the contrary, it was much higher than the 61.2 bpm reported by Eroglu et al. [41] and considerably lower than Kirk and Parker’s [37] value of 79 bpm. The HR_rest_ protocol may have an impact on the measured values; consequently, it is recommended to monitor HR when the individual is sleeping [46], although in this scenario, the participants’ consent is required.

The grand means of heart rate during work for the four examined postures ranged from 112.95 to 125.83 bpm, suggesting that the tree felling operations fall into the heavy work category. Our study results may not be directly comparable with previous research given that our focus was on tree felling and excluded further tree processing work phases. However, it is not uncommon in the study area that some forest workers who fell trees at the beginning of their shift will further process them into semifinal and final products at a later stage. Our HR_work_ grand mean range is higher than those of 97 bpm reported for forest exploitation workers in Turkey [7], 107.1 bpm reported for tree fellers in Romania [2], and nearly 112 bpm reported for pruning workers in New Zealand [37]. On the contrary, our results differentiate on the basis of the examined body posture: flexed squatting is of similar magnitude to the 122.8 bpm for felling workers in Turkey [5] and the 125 bpm reported for felling workers in Poland [40], whereas the other three postures resulted in lower heart strain. A more direct comparison can be made as follows [28]: flexed stooping resulted in the highest values with similar results (125.83 vs. 125.3 bpm), followed by stooping (117.04 vs. 121.05 bpm). In both studies, squatting resulted in close values (115.15 vs. 114.1 bpm), but this was not the case with half kneeling, which in our study was associated with the lowest HR_work_ but was the third highest in the other study (112.95 vs. 116.3 bpm). Based on these results, we verified the value ranges in most cases; however, more research is needed to explain the differences found during stooping and half kneeling.

All subjects regardless of posture had HR_work_ values within the range of 111.02 to 129.24 bpm that are characteristic of heavy work. During squatting and half kneeling, three and one subjects, respectively, had a work session that could have been described as of a moderate heart strain, not exceeding 110 bpm.

According to the GLM models, posture was the only common factor affecting HR_work_, ΔHR, and HRR. In the case of HR_work_, this finding is strongly supported by the difference among the grand means: flexed stooping resulted on average in 125.83 bpm, compared with 117.04, 115.16, and 112.95 bpm found for stooping, squatting, and half-kneeling, respectively. This order in terms of magnitude is also reported for HRR: flexed stooping exerted heart strain equivalent to very heavy work at 53.91%, compared with 45.5% during stooping, 43.92% during squatting, and 41.32% during half kneeling. The grand mean relative heart rate during work (HRR) was 43.54%, indicating heavy labor strain. This score exceeds the suggested range of 30–40% of aerobic capacity. Leszczynski and Stanczykiewicz [40] found that forest workers had higher HRR during initial thinning (48.69%) than during final thinning (41.61%) operations, whereas the respective values for tractor drivers and processor operators were much lower, reaching only 22.85% and 22.64%, respectively. Considerable differences in terms of HRR have been reported for harvesting workers (40.9%) and nursery workers (32.4%) in Turkey [41]. The HRR of choker setters (forest workers responsible for pulling out the cable and attaching logs to it during wood extraction) ranged from 44 and 46% [4], and 46% has been reported in the case of manual clearcutting in a Romanian poplar plantation [2].

The mean subject BMI in this study was 25.68 kg m^−2^, which is close to the 25.1 kg m^−2^ reported for felling workers in Turkey [5] and 24.9 kg m^−2^ for choker setters in New Zealand [46]. The lowest index value (22.6 kg m^−2^) to our knowledge was reported for forest workers in Canada [47] and the highest (27.4 kg m^−2^) for Polish forest workers [28].

In large-sample cross-sectional studies, higher BMI has typically been linked to increased workload, even in young adulthood [48], and to lower heart rate recovery index [49]. However, we found a reversed trend from the expected: subjects with a BMI of up to 25 kg m^−2^ experienced higher ΔHR by 4.21 bpm and HRR by 2.83% compared with their overweight and obese colleagues.

High BMI values suggesting overweight and obesity have been linked to reduced heat tolerance [50]. This is significant for hot and humid work settings such as the study region, emphasizing the importance of implementing heat stress mitigation measures, especially for high BMI subjects to avoid increases of their physiological strain [51].

In accordance with previous studies [52,53], Age class exerted a significant impact on both ΔRR and HRR. Although age has been reported as one of the most important factors affecting the physical workload of chainsaw operators in Turkey [53], our data show that the subjects belonging to Age class 1 (≤44.62 years) had a ΔHR of 8.84 bpm and an HRR increase of 5.74% compared with their elder colleagues. In a previous study in the same area, a similar result was reported [3]. Possible impacts of the variable type (ordinal vs. continuous) and the cut-off value used were not verified. An explanation for these unexpected results may lie in the interplay between work experience and the required time for tree felling, suggesting that more experienced tree fellers fulfilled their task in a more efficient way. A similar finding was reported for construction professions in Norway by Lunde et al. [53]. Another possible explanation may be that the older workers were in better health than their younger colleagues. This point has to be further examined in future studies.

Finally, the average age of the study subjects was 44.62 ± 6.46 years, an indication of an aging forest workforce in the study area [54]. As in many areas around the world, the lack of forest workforce may endanger the management of forested areas in the future and has to be properly addressed.

### 4.2. Suggested Ergonomic Interventions

Incorrect posture has negative effects on the spine, whereas correct posture is regarded as a prerequisite for healthy life [55]. People often tend to change their posture at work or their leisure time and adopt harmful ones due to habits they develop over time. This trend is detectable during work, especially when work tasks take place on difficult terrain and power tool operation, such as chainsaw, is involved. Generally, teaching and maintaining a proper body posture during work is basic knowledge taught in vocational training programs [56] regardless of the field of specialization. The best way to maximize productivity and ergonomic benefits is to obtain this information as soon as possible [55], and an intervention of paramount importance would be the establishment of a forest worker training system in the area that is expected to actively support the local workforce.

Two types of forest workers can be found in Iran: those hired by forest enterprises and the daily wage workers. In the first case, there is an occupational health and safety department that monitors occupational health and safety issues, occupational rights and benefits, and work accidents according to the legislation in force. The health status of the forest workers at the time of employment is recorded, and their working status is supervised by Labor Department inspectors. On the contrary, it is difficult to fulfill these provisions for daily wage workers, especially when they are employed in remote areas. There is considerable room for improvement where both worker types can benefit in the form of health surveillance initiatives. Health surveillance will help systematically monitor the workers’ health, identify potential workplace risks, and detect early signs of health problems in the workforce. Furthermore, it may facilitate the training of employees on health-related issues, a much-needed development in cases where no specialized vocational training is provided, such as in the study area. Thus, specialized seminars or training courses on maintaining good posture during work both for younger and experienced forest workers should be offered (e.g., [57]), tailor-made to their specific needs [58,59]. The value added of such training initiatives is often difficult to quantify but expands to topics beyond safety and health during work, such as the demonstration of novel equipment and working methods [60,61], reduced environmental impacts of forest operations [62], and reduced frequency and severity of accidents [1,63].

The present study has been focused on the examination of posture on the physiological strain of tree fellers. However, tree felling is only one of the multiple tasks that a forest worker has to carry out, especially in the small-scale motor–manual forest operations context. So far, research has been, in most cases, centered on tree felling, a justified decision considering the increased heart strain during this work phase [3]. At the same time, this stresses the importance of evaluating other work phases as well. During the data collection, we identified a huge potential for improvement, as the local workforce frequently adopted suboptimal postures during the various work phases. Informal conversations with the study subjects revealed that most of them suffered from MSDs, in keeping with the well-established connection between poor posture and high MSD prevalence [2,14,64]. Thus, as a first step in alleviating physiological strain, the forest entrepreneur and state authorities need to communicate the following suggestions to the local workforce: (i) avoid flexed stooping when possible and replace, when possible, with other postures during tree felling; (ii) replace unnecessary long chainsaw bars, like the ones used in the area, with shorter ones [3,65] that will help with maintaining correct posture; (iii) avoid unnecessary chainsaw operation when possible and replace with less physically demanding tools [66], and (iv) conduct work inspections that expand to the ergonomic aspects of forest work, which can prove beneficial for the health and safety of forest workers [64].

### 4.3. Strengths and Limitations of the Study

The current study is the among the few examining the impact of posture on the physiological strain of tree fellers. Building on the study of Grzwiński et al. [28], its innovational character is underlined by the analysis of four postures under real-life conditions during which 1560 trees of varying dimensions and difficulty were felled. The considerably large sample size enabled us (a) to include factors such as Age class and BMI class in the analysis and (b) to reach more accurate conclusions regarding the study area workforce.

Some weaknesses of the study should be also pointed out. Τhe most important one is the relatively limited number of subjects, which came as a consequence of the harvesting activity prohibition in natural forests in Iran for the period 2018–2023. Thus, data collection for the purpose of this study could be carried out only in plantation forests, where a small portion of the Iranian forest workforce is employed. In addition to that, some forest workers were unwilling to participate, as (a) some did not have similar previous experience and (b) some personal information was required. Furthermore, the subjects relied on their on-the-job experience rather on any systematic vocational training, which potentially introduced differences in terms of technique among them and in the resulting physiological strain. Finally, the self-reporting nature of the interview regarding the health conditions of the study participants could have contributed to misjudgments of the study participants’ health condition.

## 5. Conclusions

This study aimed to evaluate the impact of posture on physiological strain based on heart rate measurements during tree felling operations in a poplar plantation in northern Iran. Data analysis showed that tree felling fulfills the criteria of heavy work, with a strong association between heart strain and posture. Furthermore, the very popular posture of flexed stooping among the local forestry workforce is responsible for considerable increases in terms of heart strain.

The findings of our study highlighted the need for rigorous ergonomics investigations in the Iranian forest operations sector. It soon became clear that the study participants were interested in the research as it was centered on their occupational activities, and they expressed their willingness to participate in similar future endeavors. Furthermore, the lack of capital to acquire modern and also expensive forest equipment inevitably moved our focus to interventions aiming at the current level of mechanization. Due to the multifactorial character of human fatigue and the complexity of forestry operations, research on the topic should be continued and expanded to more aspects of forestry operations that will improve our insights into the topic and further elucidate local needs. Three intervention levels can be identified: state level in the form of a vocational training system for forestry professionals, local forest administrations and health authorities’ provision of helpful coursework, and forest enterprises. Ergonomic improvements are expected to improve working conditions to a considerable extent once they are properly identified and systematically implemented and supported by all involved parties. Moreover, training initiatives should be combined with more systematic health surveillance for both the workers employed by forest enterprises and the daily wage workers. Health surveillance should become a priority for forest management to support workplace cultures that incorporate monitoring and prevention as part of a workplace safety culture that values and improves the occupational health and safety of forest workers.

## Figures and Tables

**Table 1:** Study area description.

Characteristic	Value
Altitude	100–300 m
Slope	0–30%
Average Annual rainfall	989 mm
Annual average temperature range	10.7–21.3 °C
Annual average temperature	16 °C
Average Annually Humidity	82.3%
Climate classification	Very humid
Total harvested log volume in 2020–2021	7950 m^3^
Average diameter of poplar tress	30.2 cm

**Table 2:** Physical work classification according to Çaliskan and Çaglar [5].

Workload Levels	Energy Expenditure (kcal/min)	Energy Expenditure in 8 h (kcal)	Heart Rate (bpm)	Physiological Workload (%)
Resting	1.5	<720	50–60	0–10
Very light work	1.6–2.5	720–1200	60–70	10–20
Light work	2.5–6	1200–1400	70–90	20–30
Moderate work	5–7.5	2400–3600	90–110	30–40
Heavy work	7.5–10	3600–4800	110–130	40–50
Very heavy work	10–12.5	4800–6000	130–150	50–60
Unduly heavy work	>12.5	>6000	>150	>60

**Table 3:** Subject information (n = 13).

Variable (Unit)	Mean	Std. Deviation	Range
Age (years)	44.62	6.46	36–59
Height (cm)	170	0.24	156–185
Weight (kg)	74.70	11.24	62.39–96.25
Body Mass Index (kg m^−2^)	25.68	2.81	20.61–30.77
Work experience (years)	18.08	5.16	9–29

**Table 4:** Average diameter at breast height (DBH) of the 30 felled trees per body posture and average tree felling time consumption per subject and posture.

	Stoop		Flexed-Stoop		Squat		Half-Kneel	
Subject	Average DBH (cm)	Time Consumption (min)	Average DBH (cm)	Time Consumption (min)	Average DBH (cm)	Time Consumption (min)	Average DBH (cm)	Time Consumption (min)
1	36.83	3.09	36.93	3.23	36.30	3.10	36.03	3.00
2	30.33	2.65	30.40	2.69	29.63	2.61	30.50	2.61
3	32.83	2.82	33.60	2.86	33.10	2.78	32.73	2.78
4	29.17	2.57	29.80	2.59	29.17	2.55	28.73	2.50
5	31.33	2.75	31.40	2.74	31.13	2.70	31.83	2.70
6	29.83	2.63	29.27	2.60	29.97	2.54	29.73	2.50
7	31.83	2.77	31.50	2.79	31.97	2.72	31.47	2.70
8	31.33	2.74	31.27	2.76	31.30	2.68	30.73	2.69
9	32.83	2.87	32.13	2.87	32.77	2.82	31.80	2.80
10	32.17	2.80	31.60	2.83	32.00	2.76	31.97	2.76
11	34.50	3.00	34.37	3.00	34.50	2.86	34.20	2.98
12	33.67	2.93	33.90	2.96	33.63	2.89	32.77	2.87
13	38.67	3.29	38.67	3.34	38.43	3.17	37.93	3.19
Grand mean	32.72 ^a^	2.84	32.68 ^a^	2.87	32.60 ^a^	2.78	32.34 ^a^	2.78
SD	2.70	0.2	2.75	0.22	2.66	0.19	2.51	0.20

Note: Different letters denote statistically significant differences among the grand means at α = 0.05 level.

**Table 5:** Average heart rate during tree felling (HR_work_) in bpm per study subject for the examined body postures.

	Posture											
	Stoop			Flexed Stoop			Squat			Half-Kneel		
Subject	N	Mean	SD	N	Mean	SD	N	Mean	SD	N	Mean	SD
1	186	115.24 ^b^	2.82	194	129.49 ^d^	4.41	186	117.12 ^cd^	3.07	180	111.02 ^b^	2.77
2	159	115.46 ^b^	3.54	161	127.17 ^bc^	4.28	157	115.77 ^b^	3.07	157	111.36 ^bc^	3.35
3	169	113.55 ^a^	3.08	171	128.09 ^c^	4.58	169	117.16 ^cd^	2.29	167	109.29 ^a^	2.88
4	154	117.45 ^c^	3.24	156	126.78 ^bc^	3.74	153	116.95 ^cd^	2.69	150	112.97 ^de^	3.04
5	165	117.58 ^c^	3.02	164	126.98 ^bc^	3.75	162	117.88 ^de^	3.04	162	113.49 ^ef^	3.01
6	158	120.27 ^e^	3.03	156	126.20 ^b^	3.51	152	116.26 ^bc^	2.98	150	116.72 ^h^	2.91
7	166	116.04 ^b^	2.16	167	126.96 ^bc^	3.80	163	116.68 ^bc^	2.74	162	112.14 ^cd^	2.09
8	164	115.96 ^b^	2.22	165	127.17 ^bc^	3.86	161	116.50 ^bc^	2.79	162	111.69 ^bc^	2.35
9	172	116.15 ^b^	2.90	172	127.01 ^bc^	3.86	169	116.56 ^bc^	2.88	168	111.60 ^bc^	2.46
10	168	119.27 ^de^	3.31	170	127.69 ^c^	3.66	165	118.93 ^e^	3.17	166	115.31 ^g^	3.48
11	179	119.07 ^d^	2.65	180	121.19 ^a^	3.13	172	109.34 ^a^	3.48	178	115.03 ^g^	2.63
12	175	117.69 ^c^	2.80	177	121.03 ^a^	3.08	173	109.67 ^a^	3.38	172	114.32 ^fg^	2.81
13	197	117.85 ^c^	2.62	201	121.09 ^a^	3.1	190	109.57 ^a^	3.68	191	113.54 ^ef^	2.62
Total	2212	117.04 ^a^	3.39	2234	125.83 ^b^	4.71	2172	115.16 ^c^	4.49	2165	112.95 ^d^	3.42

Note: Different letters on the same line indicate statistical differences at α = 0.05. Different letters on the last row indicate statistical differences among the grand means at α = 0.05.

**Table 6:** Resting heart rate (HR_rest_), maximum heart rate (HR_max_), and relative heart rate (HRR) per study subject for the examined body postures.

			HRR (%)			
Subject	HR_rest_	HR_max_	Stoop	Flexed Stoop	Squat	Half-Kneel
1	70	179	41.50	**54.58**	43.23	37.63
2	72	173	43.03	**54.62**	43.34	38.97
3	67	180	41.19	**54.06**	44.39	37.42
4	69	184	42.13	**50.24**	41.70	38.23
5	72	161	**51.21**	**61.78**	51.55	46.62
6	73	178	45.02	**50.67**	41.20	41.64
7	72	175	42.76	**53.36**	43.38	38.97
8	65	178	45.10	**55.02**	45.58	41.32
9	76	174	40.97	**52.05**	41.39	36.33
10	68	171	49.78	**57.95**	49.45	45.93
11	65	176	48.71	**50.62**	39.95	45.07
12	62	167	**53.04**	**56.22**	45.40	49.83
13	59	184	47.08	49.67	40.46	43.63
Total	68.46	175.38	45.5 ^a^	**53.91 ^b^**	43.92 ^a^	41.66 ^a^
SD	4.79	6.46	4.09	3.44	3.43	4.22

Note: Different letters denote statistically significant differences among the grand means at α = 0.05 level. Bolded numbers suggest very heavy physiological strain.

**Table 7:** ANOVA table for the GLM examining the heart rate during work (HR_work_), the increase over the resting heart rate during tree felling (ΔHR), and the relative heart rate index (HRR).

Model	Dependent Variable	Source	SS	df	Partial η^2^	F	*p*-Value
1	HR_work_	Corrected Model	1190.129	18	0.923	16.757	0.000
		Intercept	1339.988	1	0.931	339.606	0.000
		Posture	530.471	3	0.843	44.814	0.000
		Error	98.643	25			
2	ΔHR	Corrected Model	1482.097	18	0.848	7.744	0.000
		Intercept	0.326	1	0.001	0.031	0.862
		Experience	142.937	1	0.350	13.443	0.001
		Time	69.007	1	0.206	6.490	0.017
		Posture	401.409	3	0.602	12.584	0.000
		BMI class	154.752	1	0.368	14.554	0.001
		Age class	197.970	1	0.427	18.618	0.000
		Error	265.828	25			
3	HRR	Corrected Model	1430.312	18	0.904	13.119	0.000
		Intercept	17.542	1	0.104	2.896	0.101
		Experience	291.935	1	0.658	48.196	0.000
		Time	32.534	1	0.177	5.371	0.029
		Posture	402.381	3	0.727	22.143	0.000
		BMI class	52.420	1	0.257	8.654	0.007
		Age class	33.739	1	0.182	5.570	0.026
		Error	151.430	25			

Note: Experience: Subject experience in years; Time: Time consumption during tree felling in min.

**Table 8:** Goodness-of-fit and parameter estimates for the general linear models of the study.

Model	Adjusted R^2^	Parameter	Estimate	Std. Error	t	*p*-Value	95% CI	
							Lower Bound	Upper Bound
1	0.868	Intercept	128.162	7.408	17.300	0.000	112.905	143.420
		[Posture 1]	3.873	1.447	2.677	0.013	0.893	6.853
		[Posture 2]	12.397	1.522	8.146	0.000	9.262	15.531
		[Posture 3]	2.260	1.406	1.608	0.120	−0.635	5.155
		[Posture 4]	0					
2	0.738	Intercept	−7.015	12.161	−0.577	0.569	−32.062	18.032
		Experience	0.612	0.167	3.666	0.001	0.268	0.956
		Time	47.151	18.508	2.548	0.017	9.032	85.269
		[Posture 1]	2.366	2.375	0.996	0.329	−2.526	7.259
		[Posture 2]	10.244	2.498	4.100	0.000	5.098	15.389
		[Posture 3]	1.997	2.308	0.865	0.395	−2.756	6.750
		[Posture 4]	0					
		[BMI class 1]	4.213	2.900	1.453	0.159	−1.760	10.185
		[BMI class 2]	0					
		[Age class 1]	8.842	3.023	2.925	0.007	2.617	15.068
		[Age class 2]	0					
3	0.835	Intercept	8.330	9.179	0.907	0.373	−10.575	27.234
		Experience	0.875	0.126	6.942	0.000	0.616	1.135
		Time	32.375	13.969	2.318	0.029	3.605	61.145
		[Posture = 1]	3.081	1.793	1.718	0.098	−0.612	6.773
		[Posture = 2]	11.507	1.886	6.103	0.000	7.624	15.391
		[Posture = 3]	2.311	1.742	1.327	0.197	−1.276	5.898
		[Posture = 4]	0					
		[BMI class = 1]	2.831	2.189	1.293	0.208	−1.677	7.338
		[BMI class = 2]	0					
		[Age class = 1]	5.735	2.282	2.514	0.019	1.036	10.434
		[Age class = 2]	0					

Note: Experience: Subject experience in years; Time: Time consumption during tree felling in min; Posture 1: Stooping; Posture 2: Flexed stoop; Posture 3: Squat; Posture 4: Half-kneel.

## Data Availability

Not applicable.

## References

[1] Tsioras P.A., Rottensteiner C., Stampfer K. (2014). Wood harvesting accidents in the Austrian State Forest Enterprise 2000–2009. Saf. Sci..

[2] Cheta M., Marcu M.V., Borz S.A. (2018). Workload, Exposure to Noise, and Risk of Musculoskeletal Disorders: A Case Study of Motor-Manual Tree Feeling and Processing in Poplar Clear Cuts. Forests.

[3] Arman Z., Nikooy M., Tsioras P.A., Heidari M., Majnounian B. (2021). Physiological workload evaluation by means of heart rate monitoring during motor-manual clearcutting operations. Int. J. For. Eng..

[4] Aalmo G.O., Magagnotti N., Spinelli R. (2016). Forest workers and steep terrain winching: The impact of environmental and anthropometric parameters on performance. Croat. J. For. Eng..

[5] Çaliskan E., Çaglar S. (2010). An assesment of physiological workload of forest workers in felling operations. Afr. J. Biotechnol..

[6] Dvořák J., Natov P., Natovová L., Krilek J., Kováč J. (2016). Operator’s physical workload in simulated logging and timber bucking by harvester. J. For. Sci..

[7] Eroglu H., Yilmaz R., Kayacan Y. (2015). A Study on Determining the Physical Workload of the Forest’ Harvesting and Nursery-Afforestation Workers. Anthropologist.

[8] Spinelli R., Aalmo G.O., Magagnotti N. (2014). The effect of a slack-pulling device in reducing operator physiological workload during log winching operations. Ergonomics.

[9] Staal Wästerlund D. (2001). Heat Stress in Forestry Work. Ph.D. Thesis.

[10] Ackerknecht C. (2010). Work in the forestry sector: Some issues for a changing workforce. Unasylva (Engl. Ed.).

[11] Tsioras P.A. (2012). Status and Job Satisfaction of Greek Forest Workers. Small-Scale For..

[12] Miranda H., Viikari-Juntura E., Martikainen R., Takala E.P., Riihimaki H. (2001). Physical exercise and musculoskeletal pain among forest industry workers. Scand. J. Med. Sci. Spor..

[13] Hagen K.B., Magnus P., Vetlesen K. (1998). Neck/shoulder and low-back disorders in the forestry industry: Relationship to work tasks and perceived psychosocial job stress. Ergonomics.

[14] Grzywinski W., Wandycz A., Tomczak A., Jelonek T. (2016). The prevalence of self-reported musculoskeletal symptoms among loggers in Poland. Int. J. Ind. Ergon..

[15] Gallis C. (2006). Work-related prevalence of musculoskeletal symptoms among Greek forest workers. Int. J. Ind. Ergon..

[16] Bovenzi M., Zadini A., Franzinelli A., Borgogni F. (1991). Occupational musculoskeletal disorders in the neck and upper limbs of forestry workers exposed to hand-arm vibration. Ergonomics.

[17] Rottensteiner C., Tsioras P., Stampfer K. (2012). Wood density impact on hand-arm vibration. Croat. J. For. Eng..

[18] Rottensteiner C., Stampfer K. (2013). Evaluation of operator vibration exposure to chainsaws equipped with a Kesper safety bar. Scand. J. For. Res..

[19] Tunay M., Melemez K. (2008). Noise induced hearing loss of forest workers in Turkey. Pak. J. Biol. Sci..

[20] Stewart M., Koltes K., Lehman M., St. Juliana A., Dougovito J., Pryal J. (2005). Noise Exposure Levels for Workers in the Michigan Wood Industry.

[21] Neitzel R., Yost M. (2002). Tasks-based assessment of occupational vibration and noise exposures in forestry workers. Am. Ind. Hyg. Assoc. J..

[22] Kovac J., Krilek J., Dado M., Beno P. (2018). Investigating the Influence of Design Factors on Noise and Vibrations in the case of Chainsaws for Forestry Work. FME Trans..

[23] Čavlović A.O., Beljo Lučić R., Jug M., Radmanović K., Bešlić I. (2013). Side-by-side determination of workers’ exposure to wood dust with iom and openfaced samplers. Arh. Hig. Rada Toksikol..

[24] Dimou V., Malesios C., Chatzikosti V. (2020). Assessing chainsaw operators’ exposure to wood dust during timber harvesting. SN Appl. Sci..

[25] Leszczyński K. (2014). The concentration of carbon monoxide in the breathing areas of workers during logging operations at the motor-manual level. Int. J. Occup. Med. Environ. Health.

[26] Hooper B., Parker R., Todoroki C. (2017). Exploring chainsaw operator occupational exposure to carbon monoxide in forestry. J. Occup. Environ. Hyg..

[27] Borz S.A., Iordache E., Marcu M.V. (2021). Enhancing Working Posture Comparability in Forest Operations by the Use of Similarity Metrics. Forests.

[28] Grzywiński W., Jelonek T., Tomczak A., Jakubowski M., Bembenek M. (2017). Does body posture during tree felling influence the physiological load of a chainsaw operator?. Ann. Agric. Environ. Med..

[29] Joyner M.J., Casey D.P. (2015). Regulation of increased blood flow (hyperemia) to muscles during exercise: A hierarchy of competing physiological needs. Physiol. Rev..

[30] Pheasant S., Haslegrave C.M. (2006). Bodyspace: Anthropometry, Ergonomics and the Design of Work.

[31] Peacock B., Charlton S.G., O’Brien T.G. (2002). Measurement in Manufacturing Ergonomics. Handbook of Human Factors Testing and Evaluation.

[32] Hagen K.B., Vik T., Opsahl P.A. (1993). Physical workload, perceived exertion, and output of cut wood as related to age in motor-manual cutting. Ergonomics.

[33] Arman Z., Nikkoy M., Tsioras P.A., Heidari M.J., Majnounian B. (2022). Mental Workload, Occupational Fatigue and Musculoskeletal Disorders of Forestry Professionals: The Case of a Loblolly Plantation in Northern Iran. Croat. J. For. Eng..

[34] Dimou V., Malesios C., Pispa S. (2020). Monitoring self-reported musculoskeletal symptoms in forestry operations. Int. J. For. Eng..

[35] World Health Organization Mean Body Mass Index. https://www.who.int/gho/ncd/risk_factors/bmi_text/en/.

[36] Tunceli K., Li K.M., Williams L.K. (2006). Long-term effects of obesity on employment and work limitations among US adults, 1986 to 1999. Obesity.

[37] Kirk P.M., Parker R.J. (1996). Heart rate strain in New Zealand manual tree pruners. Int. J. Ind. Ergon..

[38] Vitalis A., Pournaras N.D., Jeffrey G.B., Tsagarakis G., Monastiriotis G., Kavvadias S. (1994). Heart rate strain indices in Greek steelworkers. Ergonomics.

[39] Roy S., McCrory J. (2015). Validation of Maximal Heart Rate Prediction Equations Based on Sex and Physical Activity Status. Int. J. Exerc. Sci..

[40] Leszczynski K., Stanczykiewicz A. (2015). Workload analysis in logging technology employing a processor aggregated with a farm tractor. For. Syst..

[41] Eroglu H., Kayacan Y., Yilmaz R. (2015). Effects of Work Types and Workload on Certain Anthropometric Parameters in Forestry Workers. Anthropologist.

[42] Apud E., Bostrand L., Mobbs I.D., Strehlke B. (1989). Guide-Lines on Ergonomic Stydy in Forestry.

[43] Abeli W.S., Malisa E.J. Productivity and workload when cutting with peg and raker toothed cross cut saws. Proceedings of the International Seminar on Forest Operations under Mountainous Conditions.

[44] Melemez K., Tunay M. Investigation of Physical Work Load on Forest Harvesting Operations in Bartin-Kumluca Region. Proceedings of the XVII National Ergonomics Congress.

[45] Grandjean E. (1980). Fitting the Task to the Man: An Ergonomic Approach.

[46] Kirk P.M., Sullman M.J.M. (2001). Heart rate strain in cable hauler choker setters in New Zealand logging operations. Appl. Ergon..

[47] Dube P.A., Imbeau D., Dubeau D., Auger I., Leone M. (2015). Prediction of work metabolism from heart rate measurements in forest work: Some practical methodological issues. Ergonomic.

[48] Mänttäri S.K., Oksa J.A.H., Virkkala J., Pietilä J.A.K. (2019). Activity Level and Body Mass Index as Predictors of Physical Workload During Working Career. Saf. Health Work.

[49] Föhr T., Pietilä J., Helander E., Myllymäki T., Lindholm H., Rusko H., Kujala U.M. (2016). Physical activity, body mass index and heart rate variability-based stress and recovery in 16 275 Finnish employees: A cross-sectional study. BMC Public Health.

[50] Bastardot F., Marques-Vidal P., Vollenweider P. (2019). Association of body temperature with obesity. The CoLaus study. Int. J. Obes..

[51] Yazdanirad S., Dehghan H., Rahimi Y., Zeinodini M., Shakeriyan M. (2015). The Relationship Between Overweight and Heart Rate in Hot and Very Hot Weather Under Controlled Conditions. Health Scope.

[52] Lunde L.-K., Koch M., Veiersted K.B., Moen G.-H., Wærsted M., Knardahl S. (2016). Heavy Physical Work: Cardiovascular Load in Male Construction Workers. Inter. J. Environ. Res. Public Health.

[53] Melemez K., Tunay M. (2010). Determining physical workload of chainsaw operations working in forest harvesting. Technology.

[54] Garland J. (2018). Accident Reporting and Analysis in Forestry.

[55] Kim D., Cho M., Park Y., Yang Y. (2015). Effect of an exercise program for posture correction on musculoskeletal pain. J. Phys. Ther. Sci..

[56] Kuratorium für Waldarbeit und Forsttechnik (2019). Waldarbeitsschulen Deutschlands. Beruf Forstwirt.

[57] Harstela P. (1990). Work postures and strain of workers in nordic forest work: A selective review. Int. J. Ind. Ergon..

[58] Poje A., Potočnik I., Košir B., Krč J. (2016). Cutting patterns as a predictor of the odds of accident among professional fellers. Saf. Sci..

[59] Bordas R.M., Davis G.A., Hopkins B.L., Thomas R.E., Rummer R.B. (2001). Documentation of hazards and safety perceptions for mechanized logging operations in East Central Alabama. J. Agric. Saf. Health.

[60] Montorselli N.B., Lombardini C., Magagnotti N., Marchi E., Neri F., Picchi G., Spinelli R. (2010). Relating safety, productivity and company type for motor-manual logging operations in the Italian Alps. Acc. Anal. Prev..

[61] Aalmo G.O., Talbot B. (2014). Operator performance improvement through training in a controlled cable yarding study. Int. J. For. Eng..

[62] Tsioras P.A., Liamas D.K. (2015). Residual tree damage along skidding trails in beech stands in Greece. J. For. Res..

[63] Tsioras P.A., Rottensteiner C., Stampfer K., Tamparopoulos A.E. Slip, trip and fall accidents in a large forest enterprise. Proceedings of Safety, Reliability and Risk Analysis: Beyond the Horizon—Proceedings of the European Safety and Reliability Conference, ESREL 2013.

[64] Barbosa R.P., Fiedler N.C., do Carmo F.C.A., Minette L.J., Silva E.N. (2014). Analysis of posture in semi-mechanized forest harvesting in steep areas. Rev. Arvore.

[65] Parker R., Sullman M., Kirk P., Ford D. (1999). Chainsaw size for delimbing. Ergonomics.

[66] Apud E., Valdes S. (1995). Ergonomics in Forestry. The Chilean Case.

